# Preliminary Investigation of Injury Patterns and Preventive Behaviors Among Japanese Skateboarders: A Retrospective Cross-Sectional Study

**DOI:** 10.7759/cureus.117290

**Published:** 2026-10-01

**Authors:** Naoyuki Hayakawa, Koki Hashimoto, Rie Nakazawa, Tsuyoshi Tajika

**Affiliations:** 1 Department of Rehabilitation, Daiichi Hospital, Takasaki, JPN; 2 Rehabilitation Centre, Hidaka Hospital, Takasaki, JPN; 3 Applied Rehabilitation Sciences, Graduate School of Health Sciences, Gunma University, Maebashi, JPN

**Keywords:** ankle injuries, athletic injuries, big five personality traits, epidemiology, skateboarding

## Abstract

Objective: The popularity of skateboarding has surged in Japan following recent Olympic successes, yet comprehensive investigations into the injury patterns of Japanese skateboarders remain extremely limited. The aim of this study is to clarify the injury patterns among Japanese skateboarders and investigate their associations with competitive levels, lifestyle habits, and personality traits.

Methods: A self-administered questionnaire was completed by 65 skateboarders (recreational, n = 45; competitive, n = 20). We assessed basic attributes, injuries over the past 12 months, sporting habits, and personality traits using the Japanese Big Five Scale. Injury incidence was estimated per 1,000 exposure hours (eh).

Results: The competitive group was significantly younger, more experienced, and practiced more frequently than the recreational group (p < 0.05). Although injury prevalence was higher in the competitive group (65.0% vs. 37.8%, p = 0.060), the estimated injury incidence was higher in the recreational group (2.42 vs. 1.23/1,000 eh). After adjusting for previous sports experience using the Mantel-Haenszel method, the competitive group showed significantly higher odds of injury (pooled odds ratio (OR) = 2.68). Sprains were the most common injury type (50.9% recreational; 42.8% competitive), predominantly affecting the ankle (45.3% recreational; 47.6% competitive). Competitive skaters primarily visited hospitals (61.5%), whereas recreational skaters favored bonesetter clinics (41.1%, p = 0.029). The competitive group implemented significantly more preventive behaviors, such as warm-ups and protective gear use (p < 0.01). Conscientiousness was the most predominant personality trait in both groups; however, no significant association with injury history was observed.

Conclusions: Japanese skateboarders in this preliminary study primarily sustained lower limb sprains, particularly involving the ankle. While initial analysis adjusted for prior sports experience indicated a 2.68-fold higher odds of injury among competitive skaters, the small sample size precluded adjustments for critical demographic factors, such as age and sex, which could significantly alter these outcomes. Therefore, larger-scale studies with balanced demographic representation are necessary to draw definitive conclusions and develop robust, level-tailored injury prevention strategies.

## Introduction

The sport of skateboarding, which originated in the United States in the 1940s, is primarily categorized into Street and Park disciplines [1]. Recently, the number of participants has surged, heavily driven by the success of Japanese athletes in the Tokyo and Paris Olympics, as well as its confirmed inclusion in the 2028 Los Angeles Games [1]. Skateboarding activities take place in diverse environments, ranging from dedicated park facilities to public roads and parking lots [2]. Historically, the sport achieved global popularity following significant technical innovations in the 1970s [3]. During a subsequent skateboarding boom in Japan in the 1980s, an increase in trauma occurring in public spaces prompted local regulations and catalyzed early epidemiological research into the associated risks [4-6]. Following a temporary decline, skateboarding is currently experiencing a profound global resurgence [7]. Recent estimates indicate that the worldwide participant population exceeds 50 million, accompanied by a particularly notable increase in female athletes engaging in the sport [8,9]. Today, skateboarding is often positioned as a core urban sport alongside bicycle motocross (BMX) and parkour [10]. However, because it is fundamentally an extreme sport requiring athletes to perform complex aerial maneuvers at high speeds, it poses a continuous risk for severe trauma, such as complex fractures and head injuries [11,12]. Although medical guidelines frequently recommend the use of protective gear, the incidence of acute injuries remains stubbornly high, suggesting that safety equipment alone may be insufficient to prevent trauma [13]. Despite these well-documented risks, the majority of existing epidemiological research has relied heavily on retrospective data from Western hospital records, which may fail to capture the full spectrum of injuries managed in community settings [13]. Furthermore, the complex associations between athletes' specific practice habits, psychological factors, and the actual occurrence of injuries have not been sufficiently elucidated in the current literature [13]. As comprehensive investigations detailing these multidimensional factors in Japan remain extremely limited [1], this study aimed to clarify the actual status of injuries among Japanese skateboarders. Specifically, we sought to investigate how injury patterns are associated with lifestyle habits, personality traits, and competition-related characteristics to provide a robust foundation for tailored preventive strategies.

## Materials and methods

Participants

The study population consisted of recreational-level skateboarders (males and females in their 10s to 50s) utilizing local skateboarding facilities and competitive-level skateboarders affiliated with the All-Japan Skateboard Association (AJSA) (regardless of age and sex). The inclusion criteria were individuals with at least one year of experience who routinely participated in skateboarding at least twice a week. The exclusion criteria were as follows: (1) unreturned questionnaires; (2) respondents with major deficiencies in their answers; (3) those with less than one year of experience; and (4) elementary school students or younger respondents (Figure 1).

**Figure 1:**
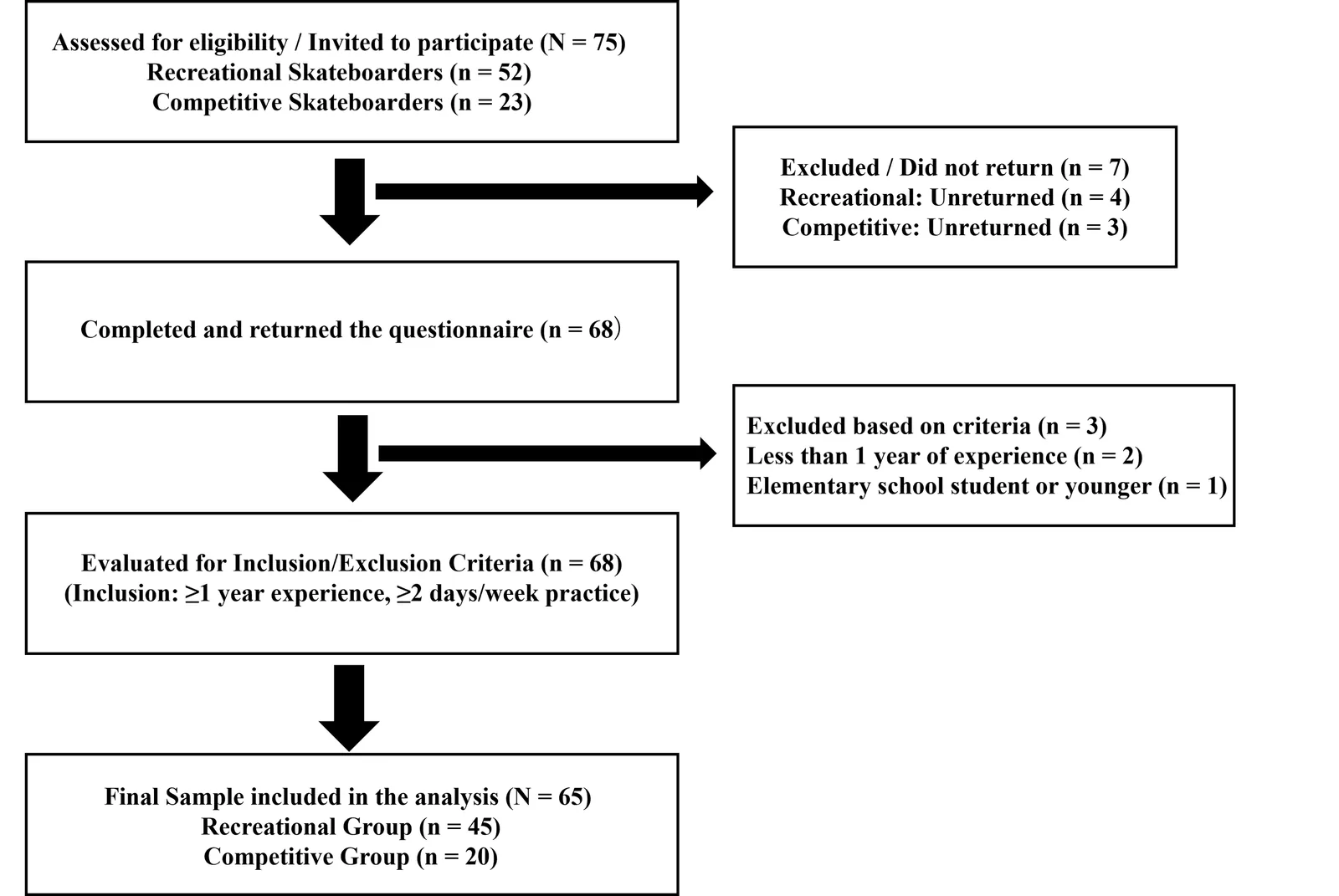
Flowchart of the participant selection process. The flowchart illustrates the study design, including the number of invited participants, response rates, application of exclusion criteria, and the final sample size for both the recreational and competitive groups.

Before conducting the study, we provided the participants and their guardians with both verbal and written information on the research purpose, methods, data privacy, and the right to withdraw. Written informed consent was obtained from all participants and their guardians before formal enrollment in the study, which was approved by the Gunma University Hospital Clinical Research Review Board (No. HS2025-122).

Questionnaire assessments

This study used a self-administered questionnaire (Appendices). The questionnaire was composed of the following four primary sections.

Basic Attributes and Competitive Background

The survey collected information on sex, age, anthropometric measurements (height and weight), medical history over the past 12 months, and skateboarding-related data. The latter included skating style, stance, years of experience, weekly frequency, average session duration, competition history, and competitive category. The stance was classified as regular (left foot forward) or goofy (right foot forward). A session was defined as any practice day on which skateboarding was performed, regardless of duration. In this study, "experience in other sports" was operationally defined as continuously participating in a sport other than skateboarding for a minimum duration of six months. This six-month cut-off was established based on the transtheoretical model of behavior change, which defines six months as the threshold for transitioning from the "action" stage to the "maintenance" stage of a physical activity habit [14,15]. Furthermore, six months of continuous practice is considered a sufficient period for athletes to acquire fundamental motor control, protective reflexes, and chronic physiological adaptations in the musculoskeletal system, which are necessary to potentially influence injury risk [16].

Sports and Lifestyle Habits

This section evaluated participants' routines related to injury prevention, including the implementation of warm-ups, cool-downs, and supplementary training. It also inquired about the use of protective gear and the practice of falling techniques. Additionally, general lifestyle habits were assessed, including the participants' interest in nutrition, smoking status, and alcohol consumption.

Injury Definition and Classification

Following Bahr et al. [17], an injury was defined as a medical attention injury occurring during skateboarding activities (sessions, practice, or competitions) that required medical intervention, evaluation, or treatment by a physician, athletic trainer, or physical therapist. Injuries with zero days of time loss were included to capture a comprehensive overview of injury patterns. In the questionnaire, participants were initially asked to report the total number of all injuries experienced in the past 12 months. However, to ensure diagnostic accuracy for the detailed anatomical and typological analyses, we exclusively utilized the subset of cases that involved specific medical interventions or professional evaluations. Based on the methodology of Bahr et al. [17], injury types were categorized into eight groups: fracture, contusion, sprain, muscle strain, dislocation, abrasion, concussion, and unknown. Affected body parts were classified into four regions: head and neck, upper extremity (shoulder, elbow, wrist, and hand/fingers), trunk (chest, thoracic spine, abdomen, and lumbosacral region), and lower extremity (hip, thigh, knee, lower leg, ankle, and foot).

Calculation of Injury Incidence

Following the methodology of Rodríguez-Rivadulla et al. [18], the estimated injury incidence per exposure unit was calculated as cases per 1,000 exposure hours (eh). As this was a cross-sectional study, the number of injuries in the 12 months preceding the survey was used as a retrospective estimate. The incidence was calculated by multiplying the number of injuries by 1,000 and dividing the result by the total exposure time (calculated as average session duration × average weekly sessions × 52 weeks per year).

Time Loss (Severity)

Time loss (absence from activity) was classified into four categories according to Bahr et al. [17]: 0, 1-7, 8-28, and > 28 days.

Injury Status Over the Past 12 Months

Participants were asked about the occurrence of injuries, affected body parts, medical consultation status, type of medical institution where treatment was received, and duration of interruption.

Personality Traits

The Japanese Big Five Scale, validated by Namikawa et al. [19], measures five dimensions: extraversion (e.g., sociability), conscientiousness (e.g., organization), neuroticism (e.g., anxiety), openness (e.g., curiosity), and agreeableness (e.g., warmth). Participants responded on a seven-point Likert scale, ranging from 1 (strongly disagree) to 7 (strongly agree).

Statistical analysis

Participants were categorized into two groups based on their activity patterns: a recreational group consisting of users of local skateboarding facilities and a competitive group consisting of members of the AJSA. Descriptive statistics were calculated for basic attributes, injury occurrence over the past year, sports and lifestyle habits, and personality characteristics. For intergroup comparisons of categorical variables, the chi-square test of independence was used, and Fisher's exact test was applied when expected frequencies were less than five. Continuous variables, specifically years of experience, weekly session frequency, and average session duration, were assessed for normality using the Shapiro-Wilk test. Based on the results, these variables were compared between the two groups using either the independent t-test or the Mann-Whitney U test. The estimated injury incidence over the past 12 months was calculated as the number of cases per 1,000 eh. Additionally, to adjust for the potential confounding effect of previous sports experience, the Mantel-Haenszel method was used to calculate the pooled odds ratio (OR) for injury occurrence between the competitive and recreational groups. All statistical analyses were performed using IBM SPSS Statistics version 29.0 (IBM Corp., Armonk, NY, USA), with the significance level set at 5%.

## Results

In the recreational group, 48 of the 52 invited individuals responded (92.3%); three were excluded (two with <1 year of experience and one elementary student), leaving 45 for analysis. In the competitive group, 20 of 23 participants responded (87.0%), with no exclusions. The overall response rate was 90.7% (68/75), resulting in a final analytical sample of 65 participants in this study.

Basic information and skateboarding-related information

Of the 65 participants, 61 were males, and four were females, with a mean age of 28.4 ± 13.3 years. The mean experience was 9.7 ± 7.7 years, with 3.3 ± 2.1 session days/week and 3.4 ± 1.7 hours/session. The competitive group was significantly younger (22.6 ± 11.3 vs. 31.0 ± 13.4 years; p = 0.025), more experienced (11.9 ± 7.1 vs. 8.8 ± 7.9 years; p = 0.004), and practiced more frequently (4.6 ± 2.2 vs. 2.7 ± 1.7 days; p = 0.003) than the recreational group. The competitive category distribution also differed significantly (p = 0.002), with all five professionals belonging to the competitive group. No significant intergroup differences were observed in sex, height, weight, medical history, or session duration (Table 1).

**Table 1:** Demographic and activity-level characteristics of the recreational and competitive groups. Results are presented as means and standard deviations (SD). P-values were calculated to compare the two groups, with statistical significance set at p < 0.05. The asterisk (*) denotes a statistically significant difference between the groups.

	Recreational group (n = 45)	Competitive group (n = 20)	p-value
Sex			
Male	41	19	1.00
Female	4	1	-
Age	31.0 ± 13.4	22.6 ± 11.3	0.025
Height	167.8 ± 7.3	168.2 ± 7.9	0.596
Weight	59.5 ± 9.0	56.9 ± 7.1	0.267
Stance			
Regular	32	14	1.00
Goofy	11	5	-
Unanswered	2	1	-
Competitive category			
Amateur	40	15	0.002^*^
Professional	0	5	-
Instructor	5	0	-
Average years of experience	8.8 ± 7.9	11.9 ± 7.1	0.004^*^
Weekly session frequency (days)	2.7 ± 1.7	4.6 ± 2.2	0.003^*^
Average session duration (hours)	3.4 ± 1.8	3.5 ± 1.5	0.697

A total of 53 participants had experience in other sports. Regarding previous sports experience, the most commonly reported sports among all participants were snowboarding (n = 17) and soccer (n = 16). Detailed information on the specific types of previous sports experiences for both the recreational and competitive groups is summarized in Table 2. A total of 26 participants had competitive backgrounds.

**Table 2:** Previous sports experience of participants in the recreational and competitive groups. Data are presented as the number of participants who reported prior experience in the respective sports. As individuals could have experience in multiple sports, the total sum of responses exceeds the total number of participants in both the recreational and competitive groups.

Sport	Number of participants (n)	Recreational group (n)	Competitive group (n)
Snowboarding	17	15	2
Soccer	16	11	5
Swimming	7	5	2
Basketball	6	2	4
Baseball	6	5	1
Road cycling	3	3	0
Track and field	2	2	0
Rugby	2	2	0
Tennis	2	2	0
Skimboarding	2	2	0
Surfing	2	2	0
Rock climbing	1	1	0
Dance	1	0	1
Table tennis	1	1	0
Softball	1	1	0
Golf	1	1	0
Kendo	1	1	0
Karate	1	0	1
Martial arts	1	0	1
Inline skating	1	1	0
Off-road motorcycling	1	1	0
Futsal	1	1	0
No previous sports experience	12	6	6

Injury occurrence over the past 12 months

The proportion of participants who experienced at least one injury in the past year was 37.8% (17/45) in the recreational group and 65.0% (13/20) in the competitive group. Although a notable trend toward a higher proportion of injured individuals was observed in the competitive group (p = 0.060), it did not reach statistical significance in the unadjusted analysis. When stratified by previous sports experience, among the 53 participants with prior sports experience, injuries were reported by nine of 14 competitive skaters and 13 of 39 recreational skaters. Conversely, among the 12 participants without prior sports experience, injuries were reported by four of six competitive skaters and four of six recreational skaters. When stratifying by previous sports experience to adjust for its potential confounding effect, the competitive group demonstrated 2.68 times higher odds of sustaining an injury compared to the recreational group (Mantel-Haenszel pooled odds ratio (OR) = 2.68). Furthermore, no significant differences were found between the two groups regarding the average number of injuries per person or the total number of injuries. Conversely, the estimated injury incidence per 1,000 exposure hours (eh) was 2.42 and 1.23 for the recreational and competitive groups, respectively (Table 3).

**Table 3:** Injury experience, medical interventions, and estimated incidence rates in the recreational and competitive groups. Numerical values are presented as the mean ± standard deviation (SD) or frequency. P-values represent the results of statistical comparisons between the recreational and competitive groups. Statistical significance was set at p < 0.05. Injury incidence was estimated per 1,000 exposure hours (eh) based on self-reported session frequency and duration. The total number of injury cases (56 and 30) represents all self-reported injury incidents. The total number of medical interventions (53 and 21) represents the subset of those injuries that specifically required medical attention or evaluation and for which detailed descriptive data were available.

	Recreational group (n = 45)	Competitive group (n = 20)	p-value
Participants who experienced at least one injury in the past year			
With injuries (n)	17	13	0.06
Without injuries (n)	28	7	-
Total number of injury cases (n)	56	30	-
Average number of injuries per capita (n)	1.24 ± 2.26	1.50 ± 2.33	0.201
Total number of medical interventions (n)	53	21	-
Number of injuries per capita (n)	1.17 ± 2.04	1.05 ± 1.23	0.327
Estimated injury incidence per 1,000 exposure hours (eh)	2.42	1.23	0.428

Types and anatomical sites of injuries over the past 12 months

Sprains were the most frequent injury type in both groups. In the recreational group (53 cases), the most common injuries were sprains (n = 27, 50.9%), followed by contusions (n = 13, 24.5%) and fractures (n = 6, 11.3%). In the competitive group (21 cases), sprains were the most frequent (n = 9, 42.8%), followed by fractures (n = 6, 28.6%) and contusions (n = 2, 9.5%). No statistically significant difference was observed in the distribution of injury types between the two groups (Table 4).

**Table 4:** Distribution and comparison of injury types between the recreational and competitive groups. Data are presented as the number of injuries and corresponding proportions (%). The p-value represents the statistical comparison of the distribution of injury types between the two groups. Statistical significance was set at p < 0.05. The total number of cases analyzed in this table (53 in the recreational group and 21 in the competitive group) represents the subset of documented injuries that involved medical intervention or evaluation, derived from the initial total of all self-reported incidents (56 and 30, respectively).

	Recreational group (n = 45)		Competitive group (n = 20)		
Types of injuries	Number of injuries (n)	Proportion (%)	Number of injuries (n)	Proportion (%)	p-value
Sprains	27	50.9	9	42.8	0.24
Fractures	6	11.3	6	28.6	-
Contusion	13	24.5	2	9.5	-
Muscle strain	3	5.7	2	9.5	-
Concussion	2	3.8	0	0	-
Dislocation	1	1.9	0	0	-
Abrasion	0	0	1	4.8	-
Others	1	1.9	1	4.8	-
Total number	53	100	21	100	-

The anatomical sites of injury were concentrated in the lower limbs in both groups. In the recreational group, 66.0% (35/53) of all injuries affected the lower limbs, with the ankle being the most common site of injury (n = 24, 45.3%). Similarly, in the competitive group, 76.2% (16/21) of injuries involved the lower limbs, with the ankle being the most frequent site (n = 10, 47.6%). These results indicate that injuries sustained over the past year were predominantly lower limb injuries, particularly involving the ankle, regardless of the competitive level (Table 5).

**Table 5:** Anatomical distribution of injury sites in the recreational and competitive groups. Data are presented as the number of injury cases and their corresponding proportions (%). Percentages were calculated based on the total number of injury cases (number = 53 in the recreational group and number = 21 in the competitive group). The total number of cases analyzed in this table (53 in the recreational group and 21 in the competitive group) represents the subset of documented injuries that involved medical intervention or evaluation, derived from the initial total of all self-reported incidents (56 and 30, respectively).

	Recreational group (Number of participants = 17)			Competitive group (Number of participants = 13)	
Injury site	Number of injuries (n)		Proportion (%)	Number of injuries (n)	Proportion (%)
Head and neck					
Head	4		7.5	0	0
Neck	4		7.5	0	0
Upper extremity					
Shoulder	1		1.9	0	0
Elbow	1		1.9	0	0
Wrist	3		5.7	3	14.3
Hand and fingers	3		5.7	1	4.8
Trunk					
Chest	1		1.9	1	4.8
Lumbosacral region	1		1.9	0	0
Lower extremity					
Thigh	1		1.9	1	4.8
Knee	1		1.9	0	0
Leg	2		3.8	1	4.8
Ankle	24		45.3	10	47.6
Foot	7		13.2	3	14.3
Others	0		0	1	4.8
Total		53	100	21	100

Medical consultation and time loss

Regarding the medical institutions visited for injuries sustained in the past year, the most frequent choice in the recreational group was bonesetter clinics (41.1%), whereas the competitive group primarily visited hospitals (61.5%). Significant intergroup differences were observed in the choice of medical institution (p = 0.029). Similarly, there was a statistically significant difference in the type of healthcare provider (p = 0.036); judo therapists were the most common in the recreational group (35.3%), whereas physicians were the most frequent in the competitive group (53.8%).

Regarding the duration of interruption from sports activities (time loss), the results for the recreational group (53 cases total) were as follows: 0 days for six cases (11.3%), 1-7 days for nine cases (17.0%), 8-28 days for six cases (11.3%), and > 28 days for five cases (9.4%), with 27 cases (51.0%) categorized as missing data. For the competitive group (21 cases total), the breakdown was as follows: 0 days for six cases (28.6%), 1-7 days for three cases (14.3%), 8-28 days for zero cases (0%), and > 28 days for five cases (23.8%), with seven cases (33.3%) having missing data. No significant difference was found between the two groups in terms of the distribution of interruption periods (p = 0.074) (Table 6).

**Table 6:** Healthcare utilization and duration of time loss following injuries in the recreational and competitive groups. Data are presented as frequencies (n). Note that the units of analysis differ across the evaluated categories: "Type of medical institution" and "Type of healthcare provider" are based on the number of participants who experienced at least one injury, whereas the "Duration of time loss" is based on the total number of individual injury cases. P-values represent the results of statistical comparisons between the recreational and competitive groups, with statistical significance set at p < 0.05. Time loss refers to the duration during which the participant was unable to perform skateboarding because of the injury. The "Duration of time loss" category includes missing or unanswered data (27 cases in the recreational group and seven in the competitive group), which are accounted for in the total number of injuries.

	Recreational group	Competitive group	
Type of medical institution	Number of participants (n)	Number of participants (n)	p-value
Hospital	2	8	0.029
Bonesetter clinic	7	2	-
Hospital and bonesetter clinic	2	0	-
No medical consultation	6	3	-
Total	17	13	-
Type of healthcare provider	Number of participants (n)	Number of participants (n)	p-value
Physician	2	7	0.036
Sports trainer	0	1	-
Physical therapist	0	1	-
Judo therapist	6	0	-
Acupuncturist	1	1	-
Unidentified	2	0	-
Unanswered	6	3	-
Total	17	13	-
Duration of time loss	Number of injuries (n)	Number of injuries (n)	p-value
0 day	6	6	0.074
1-7 days	9	3	-
8-28 days	6	0	-
>28 days	5	5	-
Missing/unanswered	27	7	-
Total	53	21	-

Training status and injury incidence

In the recreational group (n = 45), no significant differences were observed in training status, specifically years of experience, session frequency, and session duration, regardless of whether participants had experienced an injury in the past year. In contrast, within the competitive group (n = 20), participants who experienced an injury in the past year (n = 13) had significantly fewer years of experience than those who did not (n = 7). However, no significant association was found between injury occurrence and weekly session frequency or average session duration (Table 7).

**Table 7:** Training status of injured and non-injured participants within the recreational and competitive groups. Values are presented as the mean ± standard deviation (SD). The p-values indicate the results of statistical comparisons between those with and without injuries within each group. Asterisk (*) denotes statistical significance (p < 0.05).

	Recreational group (n = 45)			Competitive group (n = 20)		
	With injury (n = 17)	Without injury (n = 28)	p-value	With injury (n = 13)	Without injury (n = 7)	p-value
Average years of experience	9.8 ± 9.6	8.3 ± 6.7	1	11.2 ± 8.5	13.0 ± 3.5	0.028*
Weekly session frequency (days)	3.4 ± 2.0	2.3 ± 1.5	0.065	5.0 ± 2.1	3.8 ± 2.3	0.353
Average session duration (hours)	3.8 ± 1.6	2.9 ± 1.4	0.145	3.6 ± 1.3	3.3 ± 1.8	0.602

Sporting and lifestyle habits

The analysis of the relationship between injury occurrence over the past year and sporting/lifestyle habits revealed that the competitive group implemented preventive behaviors significantly more frequently than the recreational group. Specifically, the competitive group showed significantly higher implementation rates for three items: warm-ups (p = 0.007), supplementary training (p = 0.009), and use of protective gear (p < 0.01). Of particular note, 85.0% (17/20) of the competitive group wore protective gear, whereas only 6.7% (3/45) of the recreational group did. In contrast, no significant associations were observed between competitive levels and the practice of cool-downs or falling techniques, or with lifestyle habits, including interest in nutrition, smoking, and alcohol consumption (Table 8).

**Table 8:** Sporting and lifestyle habits in the recreational and competitive groups. Values represent the frequency. P-values indicate the results of statistical comparisons between the recreational and competitive groups. Asterisk (*) denotes statistical significance (p < 0.05). Note: For the "Implementation status of cool-downs," the recreational group total was adjusted to 45 (12 with, 32 without, 1 unidentified). For "Smoking status" and "Drinking status," calculations were limited to adult participants (age ≥20); therefore, the sums do not equal the total group number.

	Recreational group (n = 45)	Competitive group (n = 20)	p-value
Implementation status of warm-ups			
With (n)	22	17	0.007*
Without (n)	23	3	-
Implementation status of cool-downs			
With (n)	12	5	1.00
Without (n)	32	15	-
Unidentified	1	0	-
Implementation status of supplementary strength training			
With (n)	6	11	0.009
Without (n)	39	9	-
Use of protective gear			
With (n)	3	17	p<0.001*
Without (n)	42	3	-
Practice of cooldowns or falling techniques			
With (n)	7	2	0.706
Without (n)	36	18	-
Unidentified	2	0	-
Interest in nutrition			
I care about it. (n)	32	13	0.299
I care, but I'm not taking any action. (n)	6	1	-
I don't care. (n)	7	6	-
Smoking status among adults (age ≥20)			
Current smoker	15	1	0.428
Former smoker	6	1	-
Non-smoker	10	3	-
Drinking status among adults (age ≥20)			
Drinker	20	3	1.00
Non-drinker	11	2	-

Personality traits

The distribution of the Big Five personality traits was compared between the recreational (n = 45) and competitive (n = 20) groups. In both groups, conscientiousness was the most predominant trait (64.4% and 75.0%, respectively), followed by neuroticism (20.0% and 25.0%). A 2×5 chi-square test, excluding extraversion due to zero frequency in both groups, revealed no significant difference in the distribution between the two groups (p = 0.478). Furthermore, no significant associations were observed between injury history in the past year and personality traits in either group (Table 9).

**Table 9:** Big Five personality traits of injured and non-injured participants in the recreational and competitive groups. Values represent the number of participants. P-values indicate the results of statistical comparisons between the injury and non-injury subgroups within the category. Statistical significance was set at p < 0.05. Categories with zero counts in both groups (e.g., extraversion) were excluded from the statistical calculations.

	Recreational group (n = 45)			Competitive group (n = 20)		
Big Five personality traits	With injury (n = 17)	Without injury (n = 28)	p-value	With injury (n = 13)	Without injury (n = 7)	p-value
Neuroticism	4	5	0.51	4	1	0.613
Extraversion	0	0	-	0	0	-
Openness	1	3	-	0	0	-
Agreeableness	1	0	-	0	0	-
Conscientiousness	11	18	-	0	0	-
Neuroticism + conscientiousness	0	2	-	9	6	-

## Discussion

This study clarified the characteristics of injuries (type, site, and severity) sustained by Japanese skateboarders over the past year through a questionnaire survey while investigating their associations with practice habits, competitive profiles, sporting and lifestyle habits, and personality traits. The analysis revealed distinct differences in injury patterns between the recreational and competitive groups. Specifically, the unique characteristics of each group were underscored in terms of estimated injury incidence per exposure hour, medical consultation behavior, and distribution of injury sites.

The study participants (61 males and 4 females) reflected the male-dominated sex distribution in skateboarding. Although younger than the recreational group, competitive skaters had more experience and higher session frequency, suggesting an earlier start and more consistent practice. Notably, exposure volume, frequency, and duration may act as confounders when comparing the injury status across levels. While crude annual injury rates only trended higher in the competitive group (p = 0.060), adjusting for prior sports experience revealed that competitive skaters had approximately 2.68-fold higher odds of injury. This suggests that the intense physical demands and complex maneuvers inherent to competitive skateboarding carry a substantially higher risk of acute injury, independent of a skater's general athletic background. Although the initial lack of statistical significance in the unadjusted data might also stem from a small sample size or retrospective recall bias, our stratified analysis highlights the true underlying risk, warranting future prospective studies to confirm these findings.

Conversely, the estimated injury incidence per 1,000 eh was higher in the recreational group. Despite higher exposure, competitive skaters engaged in significantly more preventive behaviors (e.g., warming up, training, and gear use), which likely reduced their injury rates per unit of exposure. In contrast, a higher incidence of recreational skaters may reflect lower technical proficiency, risk perception, and self-protective behaviors.

Sprains were the most frequent injury in both groups (recreational: 50.9%; competitive: 42.8%), followed by fractures and contusions, suggesting common injury mechanisms such as falls and landings, regardless of the level. The ankle was the primary injury site, with a significant concentration in the lower limbs. This aligns with Rodríguez-Rivadulla et al. [18], who reported that recreational injuries primarily affected the lower limbs (69.7%), particularly ligamentous ankle sprains (39.3%), which have a high (70.1%) recurrence rate [18]. These injuries likely stem from high landing impacts and repetitive inversion/eversion stresses on uneven surfaces, leading to residual instability and chronic recurrence. Our findings underscore the importance of preventive measures, including ankle strengthening, balance training, and use of protective gear.

Regarding injury severity classification based on the duration of activity withdrawal, although no statistically significant difference was observed between the two groups, the competitive group showed a tendency toward higher severity, with a larger proportion of cases requiring long-term withdrawal exceeding 28 days. Previous research [3,18] has also reported that approximately half of the injured skateboarders experienced severe injuries, mainly involving ankle sprains/contusions and wrist fractures. Additionally, Keilani et al. reported that 60% of those injured while skateboarding were examined by a physician [20]. The findings of the present study suggest that a certain number of severe injuries occur irrespective of competitive level, supporting the importance of appropriate initial management and stepwise return-to-sport programs, especially for the competitive group involving high-load practice.

Significant intergroup differences were observed in the choice of medical facilities and healthcare providers. The competitive group predominantly visited hospitals and consulted physicians, likely prioritizing accurate diagnoses for return-to-sport decisions and managing more severe injuries (absences > 28 days). Conversely, the recreational group favored bonesetter clinics because of accessibility factors, such as flexible hours and convenience, resulting in higher utilization of Judo therapists. These divergent healthcare-seeking behaviors may also reflect the higher prevalence of relatively mild cases within the recreational group.

The significantly higher session frequency in the competitive group suggests that injury occurrence depends on exposure volume as well as trick difficulty. However, the lack of a significant difference in annual injury rates between the groups indicates that higher exposure does not inherently increase the risk. This suggests that preventive behaviors, such as warming up and using protective gear, effectively offset the increased exposure in the competitive group. Conversely, the higher injury incidence in the recreational group, despite lower exposure, may stem from insufficient preparation and lower technical proficiency. Our finding that less-experienced skaters were more prone to injury aligns with Shuman et al. [13]. As years of experience do not always reflect technical skill [18], multifaceted risk assessments incorporating technical proficiency, exposure, and preventive behaviors are essential.

Regarding sports habits, the competitive group showed significantly higher implementation rates for warm-ups (p = 0.007), training (p = 0.009), and use of protective gear (p < 0.01). While preventive behaviors are systematically promoted in the competitive group through tournament and facility regulations, environmental factors may lead to disparities in implementation rates within the recreational group. Shuman et al. [13] also noted a tendency among beginners to avoid protective equipment because of its appearance. As protective gear alone is insufficient to prevent injuries, it is essential to enhance educational support, including instruction on falling techniques, to reduce the risk of upper limb fractures. The high level of preparation in the competitive group aligns with the findings of Rodríguez-Rivadulla et al. [18], suggesting efforts aimed at injury prevention or performance enhancement; however, further details remain a subject for future study.

Finally, no significant association was found between lifestyle habits (health-conscious diet, smoking, and drinking) and injury experience, which may be attributed to the limited scope of the indicators used or the specific cultural background of the sports. The Big Five analysis showed no significant associations between personality traits and injury history or competition level. While both groups exhibited high conscientiousness, we found no elevated extraversion or openness, contrary to Goldberg [21], who linked these traits to extreme sports participation. This discrepancy may stem from a sampling bias toward safety-conscious participants or the predominant influence of external factors, such as practice culture and technical proficiency. Nevertheless, our findings remain consistent with those of Yang et al. [22], suggesting that personality may indirectly affect injury prevention through behaviors such as preparation and self-management. Future longitudinal research should employ skateboarding-specific scales to verify mediation processes (e.g., personality traits, preventive behaviors, and injury occurrence).

This study had several limitations. First, as this study employed a cross-sectional questionnaire survey design, it was not possible to establish causal relationships between injuries and their associated factors in this study. Second, because injury history and exposure hours were based on retrospective recall over the past 12 months, the potential for recall bias and inaccuracies in calculating estimated incidence per 1,000 exposure hours cannot be entirely excluded. Future research should employ prospective injury surveillance systems to capture real-time data on both acute trauma and overuse injuries. Third, the relatively small sample size, particularly the limited number of 20 participants in the competitive group, limits the statistical power of our analysis. A post-hoc power analysis was performed using G*Power version 3.1.9.7 (Heinrich Heine University Düsseldorf, Düsseldorf, Germany) to evaluate the statistical power for the comparison of injury prevalence between the recreational (37.8%) and competitive (65.0%) groups, indicating that the observed trend (p = 0.060) might reach significance with a larger sample size. Furthermore, an a priori power analysis using G*Power suggested that to detect a significant difference in injury prevalence between the two groups (based on the observed proportions of 37.8% and 65.0%) with a statistical power of 0.80 and an alpha level of 0.05, a total sample size of at least 102 participants (approximately 51 per group) would be required. The current sample size (n = 65) was therefore insufficient to reach definitive conclusions, and the observed trend (p = 0.060) should be validated in larger, adequately powered prospective cohorts. Another important limitation of this study is the inability to fully adjust for all potential confounding factors. Although our statistical analysis revealed significant differences in basic characteristics, such as age, between the recreational and competitive groups, the limited overall sample size (n = 65, with only 20 participants in the competitive group) precluded the use of comprehensive multivariable regression models or a strict 1:1 matching design. Implementing a case-control matching approach would have drastically reduced the sample size, inevitably introducing severe selection bias and compromising statistical power. Consequently, to capture a preliminary, real-world descriptive snapshot of Japanese skateboarders, we maintained an unadjusted cross-sectional design for several outcome measures. Therefore, the observed associations, particularly regarding injury incidence and preventive behaviors, may be partially confounded by unadjusted variables such as age, years of experience, and individual characteristics. Readers should interpret these findings with appropriate caution. Future research must involve larger prospective cohorts or matched case-control designs to allow for rigorous multivariable adjustments and the establishment of clear causality. Fourth, a high rate of missing data was observed regarding the duration of activity withdrawal (time loss), particularly among recreational skaters. This suggests that participants may have found it challenging to quantify the interruption period for minor injuries that did not lead to a complete cessation of skating, indicating a need for more granular severity scales in future questionnaires. Fifth, because the definitions of the recreational and competitive groups were based on affiliation and activity patterns, they may not fully capture the diversity of individual skill levels or specific competition characteristics. Sixth, the frequent use of bone setters (Judo therapists) by recreational skaters reflects a unique pattern of healthcare-seeking behavior in Japan. While this provides insight into domestic patterns, the inclusion of non-physician consultations may affect the comparison of injury severity with international studies that primarily utilize emergency department or physician-based records. Seventh, while the Big Five Scale is a validated instrument for general personality traits, it may lack the specific sensitivity required to capture risk-taking behaviors or psychological resilience unique to extreme sports athletes. Future investigations should incorporate sport-specific psychological metrics to better elucidate the relationship between personality and injury risk. Finally, details regarding trick difficulty, types of obstacles, and lifestyle habits collected through open-ended responses were not sufficiently quantified, and certain information had to be excluded from analysis. Therefore, future research should implement prospective cohort studies or continuous injury monitoring to facilitate more detailed and multifaceted investigations, including factors such as exposure time, trick difficulty, practice environments, and help-seeking behaviors.

## Conclusions

Injuries among Japanese skateboarders primarily involved lower-limb sprains, with the ankle most frequently affected. While our preliminary data suggest a higher overall prevalence of injury in the competitive group and a higher estimated injury incidence rate relative to exposure time among recreational skaters, these findings must be interpreted with caution. Due to the small sample size, we were unable to adequately adjust for critical confounding factors such as age and sex, which could significantly alter these outcomes. Therefore, larger-scale studies adjusting for these demographic variables are required to draw definitive conclusions and to develop robust, level-specific injury prevention strategies focusing on the lower extremities.

## Appendices

**Table 10:** The full self-administered questionnaire used in this study.

Section	Question items	Response options/Format
1. Consent & Basic Attributes	Informed consent confirmation	Checkbox for agreement
-	Sex	Male / Female
-	Age, Height, Weight	Free text (years, cm, kg)
-	Current medical history	None / Yes (Hypertension, Diabetes, Dyslipidemia, Heart disease, Cerebrovascular disease, Respiratory disease, Mental illness, Other)
-	Past medical history	None / Yes (Same options as above)
-	Other sports experience	None / Yes (Specify sport and duration)
2. Skateboarding Profile & Occupational Status	Skateboarding style	Street / Park / Recreation / Other
-	Basic stance	Regular / Goofy
-	Years of experience	Free text (Years and months)
-	Weekly session frequency	1 to 7 days per week
-	Average session duration	Free text (Hours and minutes)
-	Starting age	Free text (Years)
-	Competition experience	None / Yes (Specify frequency per year)
-	Competition awards	None / Yes (Specify highest rank)
-	Current category (Multiple selection)	Professional / Amateur / Instructor
-	Occupation	Company employee, Civil servant, Part-time, Self-employed, Student, Housewife, Unemployed, Other
3. Sports and Lifestyle Habits	Implementation of warm-ups	No / Yes (Specify content, frequency, duration)
-	Implementation of cool-downs	No / Yes (Specify content, frequency, duration)
-	Supplementary training for injury prevention/performance	No / Yes (Specify content, frequency, duration)
-	Use of protective gear	No / Yes (If yes, specify type: Helmet, Elbow pad, Wrist pad, Knee pad, and timing of use)
-	Practice of falling techniques	No / Yes (Specify content)
-	Interest in nutrition	Do not care / Care but take no action / Care and take action (Specify)
-	Smoking status (Age 20+)	Never / Former / Current (Specify type and amount)
-	Drinking status (Age 20+)	Non-drinker / Drinker (Specify frequency, type, and amount)
-	Drinking alcohol during skateboarding	Often / Sometimes / Rarely / Never / Prefer not to say
4. Injury Status Over the Past 12 Months	Current injury or pain	No / Yes (Specify location and diagnosis)
-	Injuries in the past 1 year	No / Yes (Specify number of incidents)
-	Type of injury (Multiple selection)	Concussion, Fracture, Contusion, Sprain, Muscle strain, Dislocation, Abrasion, Unknown
-	Affected anatomical site	Head/Neck, Upper extremity, Trunk, Lower extremity, Unspecified, Multiple, Other
-	Medical consultation	No / Yes (Specify diagnosis, Institution type: Hospital, Clinic, Bonesetter, Other)
-	Type of healthcare provider	Physician, Trainer, Physical Therapist, Judo Therapist, Acupuncturist, Other
-	Obstacle/Feature at the time of injury	Flat, Ledge, Hubba, Manual pad, Rail, Down rail, Pyramid, Bank, Gap/Stairs/Kicker, Ramp, Box, Other
-	Situation and trick name	Free text
-	Duration of time loss (Interruption period)	0 days / 1-7 days / 8-28 days / >28 days
5. Personality Traits	Extraversion: Untalkative, Sociable, Talkative, Extroverted, Cheerful	7-point Likert scale (1: Strongly agree - 7: Strongly disagree)
-	Conscientiousness: Irresponsible, Sloppy, Leaving things to chance, Lazy, Organized, Rash, Meticulous	7-point Likert scale (1: Strongly agree - 7: Strongly disagree)
-	Neuroticism: Prone to anxiety, Worrisome, Timid, Nervous, Depressed	7-point Likert scale (1: Strongly agree - 7: Strongly disagree)
-	Openness: Versatile, Progressive, Original, Quick-witted, Broad interests, Curious	7-point Likert scale (1: Strongly agree - 7: Strongly disagree)
-	Agreeableness: Short-tempered, Hot-tempered, Gentle, Tolerant, Self-centered, Kind	7-point Likert scale (1: Strongly agree - 7: Strongly disagree)
